# The effectiveness of traditional Japanese medicine Goshajinkigan in irradiation-induced aspermatogenesis in mice

**DOI:** 10.1186/s12906-019-2786-z

**Published:** 2019-12-11

**Authors:** Kumpei Takahashi, Kenta Nagahori, Ning Qu, Miyuki Kuramasu, Yoshie Hirayanagi, Shogo Hayashi, Yuki Ogawa, Naoyuki Hatayama, Hayato Terayama, Kaori Suyama, Shuichi Hirai, Kou Sakabe, Masahiro Itoh

**Affiliations:** 1 Department of Anatomy, Tokyo Medical University, Tokyo, 160-8402 Japan; 2 Department of Anatomy, Division of Basic Medical Science, Tokai University School of Medicine, Kanagawa, 259-1193 Japan; 3 Department of Anatomy, Aichi Medical University, 1-1 Yazakokarimata, Nagakute, Aichi 480-1195 Japan

**Keywords:** Traditional Japanese medicine, Aspermatogenesis, Irradiation, Anti-sperm antibody; Sertoli tight junctions

## Abstract

**Background:**

Infertility and gonadal dysfunction are well known side-effects by cancer treatment in males. In particularly, chemotherapy and radiotherapy induced testicular damage, resulting in prolonged azoospermia. However, information regarding therapeutics to treat spermatogenesis disturbance after cancer treatment is scarce. Recently, we demonstrated that Goshajinkigan, a traditional Japanese medicine, can completely rescue severe busulfan-induced aspermatogenesis in mice. In this study, we aimed to detect the effects of Goshajinkigan on aspermatogenesis after irradiation.

**Methods:**

This is animal research about the effects of traditional Japanese medicine on infertility after cancer treatment. C57BL/6 J male mice received total body irradiation (TBI: a single dose of 6Gy) at 4 weeks of age and after 60 days were reared a Goshajinkigan (TJ107)-containing or TJ107-free control diet from day 60 to day 120. Then, two untreated females were mated with a single male from each experimental group. On day 60, 120 and 150, respectively, the sets of testes and epididymis of the mice in each group after deep anesthetization were removed for histological and cytological examinations.

**Results:**

Histological and histopathological data showed that 6Gy TBI treatment decreased the fertility rate (4/10) in the control diet group; in contrast, in the TJ107-diet group, the fertility rate was 10/10 (*p* < 0.05 vs. 6Gy group). Supplementation with TJ107 was found to rescue the disrupted inter-Sertoli tight junctions via the normalization of claudin11, occludin, and ZO-1 expression and reduce serum anti-germ cell autoantibodies.

**Conclusions:**

These findings show the therapeutic effect on TBI-induced aspermatogenesis and the recovering disrupted gonadal functions by supplementation with TJ107.

## Background

Chemotherapy and radiotherapy are commonly used for cancer treatment, but side effects include temporary or permanent infertility. A known fact is that among the most radiosensitive organs testis is one of them and patients receiving 1.4–2.6 Gy direct testicular irradiation the spermatogenesis cannot be recovered [1]. Gonadal toxicity is associated with total body irradiation (TBI) which is often used as conditioning for transplantation of bone marrow. Around 99.5% of men as reported by previous studies experience permanent infertility who receive12Gy TBI [2] and decrease in spermatogenesis in the seminiferous tubules of mice is caused by lower TBI doses as 5–6 Gy [3, 4]. Mechanism reported through previous studies for infertility in irradiated rodent testes is germ cell-apoptosis [5–7]. Lipid peroxidation in the cellular membrane is caused by the generation of reactive oxygen species (ROS) which causes deleterious effects of irradiation in biological systems and thereby causing DNA damage in immature germ cells [8, 9]. Some studies reported that suppressed oxidative stress or downregulate the increased amount of ROS can replace testicular function in experimental animal models [10–12]. Additionally, in three-month-old rats activation of caspase-3, concomitant with increased expression of caspase-8 and decreased expression of caspase-9 induces germ cell-apoptosis [7].

Young and long-term cancer survivors are increasing as the results of early diagnosis and successfully anticancer treatments. Therefore, the strategy for disease management has subsequently changed from cure at any cost to that the quality of life became increasingly important. Currently, the most effective and established avenue of reserving fertility in young cancer survivors, such as the cryopreservation and transplantation of gonadal tissue, are still experimental [13–15]. Therefore, many experiments including hormonal treatment [16–18] and vitamin treatment [19, 20] have been conducted to develop methods to prevent or cure testicular damage after cancer treatment. However, effective treatment has not been established and male infertility after radiotherapy is now an intractable disease.

To increase testosterone levels and improve fertility in recent time, herbal medicines have been proposed [21–24] and number of herbs have shown positive effects on the improvement of semen parameters [25, 26]. Furthermore, synergistic effects and multiple biological functions of polyherbal formulation are of vast advantages over single herbal formulation. Goshajinkigan (TJ107), is an herbal medicine composed of 10 herbal drugs and widely used to treat lower urinary tract symptoms, numbness, lower back pain, and chemotherapy-induced neuropathy in Japan [27–30]. Indeed, the therapeutic mechanism of TJ107 have been well investigated in neuropathic pain and the studies demonstrated that TJ107 can improve mitochondrial function and can decrease the expression of tumor necrosis factor-α (TNFα) which is a principal mediator in pro-inflammatory processes cytokine in muscle [31, 32]. Furthermore, component elements of TJ107 such as Rehmannia Root, Cornus Fruit, Plantago Seed, Moutan Bark, and Cinnamon Bark have been reported the anti-inflammatory effects preciously [33–38]. The exact criteria for its use have not been established yet as the molecular mechanism of TJ107 remains unclear. Recently, we found that TJ107 can completely restore aspermatogenesis after busulfan treatment in mice [39]. Administering TJ107 can increase the germ cell-proliferation and normalize germ cell-apoptosis, such as the expressions of apoptotic-genes of *Fas* and *Caspase8* in busulfan-treated mice [28]. The aim of this study was to determine the effects of TJ107 on recover spermatogenesis after irradiation treatment in mice and to develop an effective method to minimize or reverse the gonadal toxicity associated with cancer treatment.

## Methodology

### Animals

Male C57BL/6j mice (4-week-old, weighting 16–20 g) were purchased from SLC (Shizuoka, Japan) and maintained in the Laboratory Animal Center of Tokyo Medical University. Animals were housed at 22–24 °C with 50–60% relative humidity and a 12-h light–dark cycle.

### Preparation TJ107 diet

As per the method described previously, the diet for TJ107 was prepared [39]. TJ107 (extract granules in powdered form; No. 2120107030 and 2,130,107,030) was manufactured by Tsumura & Co. (Tokyo, Japan) according to Japanese and International manufacturing guidelines. The constituents of TJ107 are listed in Table 1. Briefly, the TJ107 diet was prepared as mouse standard diet (Oriental Yeast Co., Ltd. (Tokyo, Japan); 23.1% crude protein [w/w], 5.1% crude fat, 5.8% crude ash, 2.8% crude fiber, and 55.3% nitrogen-free extract and mineral mixture) containing 5.4% (w/w) TJ107 extract.

**Table 1:** Constituents in 7.5 g of TJ107 (JP: The Japanese Pharmacopoeia)

Herbs	Scientific names	weight
JP Rehmannia Root	(Rehmannia glutinosa Liboschitz)	5.0 g
JP Achyranthes Root	(Achyranthes bidentage Blume)	3.0 g
JP Cornus Fruit	(*Cornus officinalis* Sieb. et Zucc.)	3.0 g
JP Dioscorea Rhizome	(*Dioscorea batatas* Decaisne)	3.0 g
JP Plantago Seed	(*Plantago asiatica*)	3.0 g
JP Alisma Rhizome	(Alisma orientale Juzep)	3.0 g
JP Poria Sclerotium	(Poria cocos Wolf)	3.0 g
JP Moutan Bark	(*Paeonia suffruticosa* Andrews)	3.0 g
JP Cinnamon Bark	(*Cinnamomum cassia* Blume)	1.0 g
JP Powdered Processed Aconite Root	(*Aconitum carmichaelii* Debeaux)	1.0 g

### Experiment design

Mice were irradiated with 6Gy using a 60Co gamma ray unit (MBR-1520A-TWZ; HITACHI KE Systems Ltd., Tokyo, Japan). A single dose of TBI was administered without anesthesia.

**Fig. 1:**
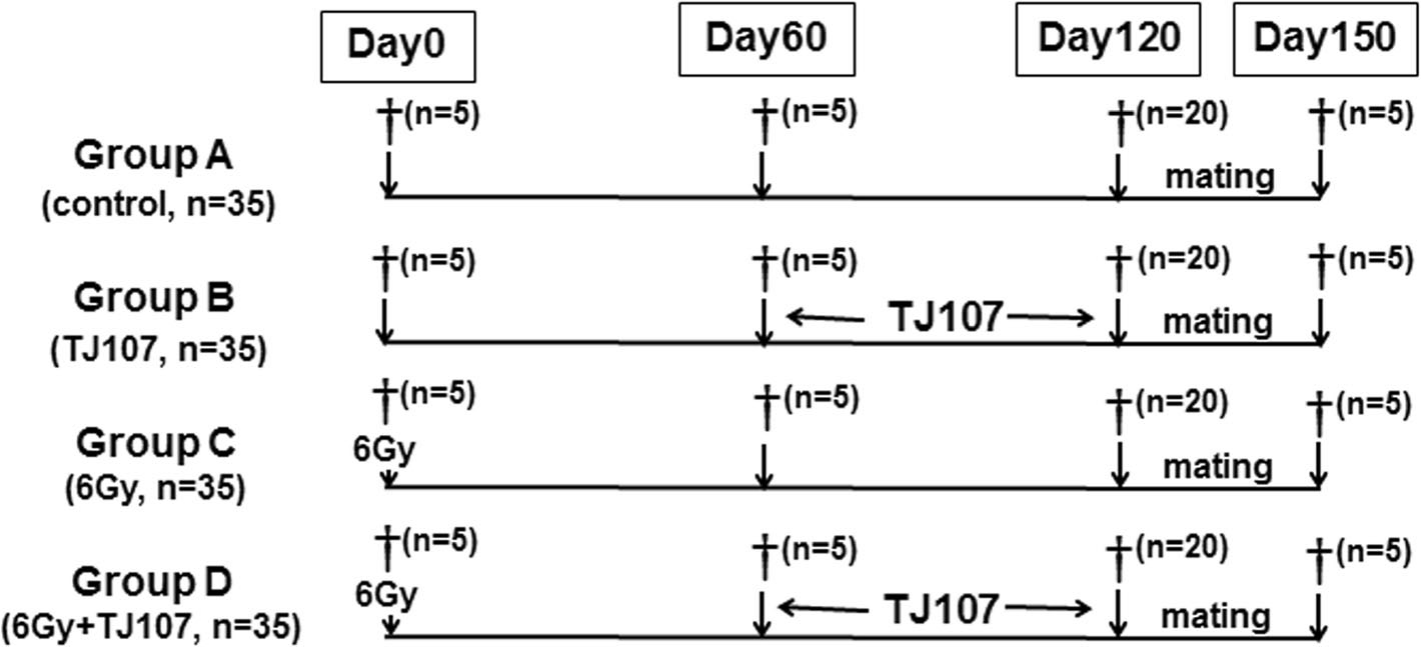
Application of treatment as per the time schedule to the mice in each group

The division of the experimental mice was into 4 groups as: Group A (normal male mice fed a standard diet to day 120 = control group; *n* = 35), Group B (normal male mice fed a standard diet to day 60 and then fed a TJ107-diet to day 120 = TJ107 group; n = 35), Group C (male mice receiving a single 6Gy dose of TBI and fed a standard diet to day 120 = 6Gy group; *n* = 35), and Group D (male mice receiving a single 6Gy TBI dose and fed a standard diet to day 60, and then fed a TJ107-diet to day 120 = 6Gy + TJ107 group; *n* = 35). Figure 1 shows the time schedule of treatment applied to the mice in each group. All of mice were offered a standard laboratory water. The general conditions including food intake and body weight were recorded for all mice at 10-day intervals from day 60 to day 120. As per the guidelines of the National Institutes of Health, all the experimental protocols in the present study were carried and were approved by the Tokyo Medical University Animal Committee (Animal ethics approval No. S-27001).

On day 60, mice in each group (*n* = 5), respectively, were terminated individually by deeply anesthetized with pentobarbital (65 mg/kg body weight) [39–42] followed by cardiac puncture for blood collection. Serum was separated by centrifugation and the serum samples from individual mice were stored at − 80 °C until used. The testes were removed for gravimetry for histological examination and epididymides were removed for epididymal spermatozoa counts.

The mice were cross-mated on day 120 from each group (*n* = 5) with untreated female C57BL/6 J mice (8–10 weeks of age; SLC, Shizuoka, Japan) at a ratio of 1:2 (male-to-female) in a cage until day 150, to assess in vivo fertilization, after which the fertility rate was determined for each group. Simultaneously, the remaining mice from each group (*n* = 20) were terminated individually on day 120 by anesthetized with 65 mg/kg body weight pentobarbital followed by cardiac puncture for blood collection. Serum was separated by centrifugation and the serum samples from individual mice were stored at − 80 °C until used. The testes and epididymides were immediately removed for histological and cytological examinations.

### Histological examination and histopathological assessment of testis

The testes of mice (*n* = 5) in each group were fixed with Bouin’s solution and embedded in plastic in whole (Technovit7100; Kulzer & Co., Wehrheim, Germany). Sections (5 μm) were obtained at 15–20 μm intervals and stained with Gill hematoxylin and 2% eosin Y (Muto PC, Tokyo, Japan) for histological examination by light microscopy observation.

The testes of mice (*n* = 5) of each group were placed in OCT compound (Miles Laboratories, Naperville, IL, USA) and stored at − 80 °C. Sections of 6 μm were incubated for 20 min with Block Ace (Yukijirushi, Hokkaido, Japan) at room temperature (RT) and then incubated with rabbit anti-mouse Ki67 monoclonal antibodies (Abcam, Cambridge, United Kingdom; 1:1000 dilution) for 2 h. After washing the sections were incubated for 30 min with goat anti-rabbit IgG (Vector, Burlingame, CA, USA). Vectastain Elite ABC Reagent (Vector Laboratories, CA, USA) with 0.05% 3,3′-diaminobenzidine-4HCl (DAB, Nickel Solution) and 0.01% H_2_O_2_ as the chromogen was used to visualize proliferation cells.

To investigate germ cell-apoptosis, TUNEL was performed pursuant the protocol described previously [43]. Paraffin sections (5–6 um) from mice of each group (n = 5) were cut onto silane-coated glass slides, dewaxed with toluene, and rehydrated in an ethanol series. The sections were treated with 5μg/ml of proteinase K in PBS at 37 °C for 15 min after washing with PBS. Then the sections were rinsed once with deionized distilled water and the commercially available kit (Apop Tag Plus Peroxidase In Situ Apoptosis Detection Kit; Serologicals Corporation, NY, USA) was used for detection of the 3′-OH end of DNA. The sections were treated with 3% H_2_O_2_ in PBS for 5 min at room temperature to block endogenous peroxidase activity. The sections were incubated with a mixture of terminal deoxynucleotidyl transferase (TdT) and digoxigenin-labelled dideoxy nucleotide in a humidified chamber at 37 °C for 1 h. After reaction with stop buffer for 10 min, the sections were incubated with an anti-digoxigenin peroxidase conjugate for 30 min. Peroxidase activity was detected by exposing the sections to a solution containing 0.05% DAB.

### Detection of serum anti-germ cell antibodies

For the detection of serum anti-germ cell antibodies, normal testes from normal 8-week-old mice were put in OCT compound (Miles Laboratories, IL, USA) and then frozen in liquid nitrogen and stored at − 80 °C until use. Sections of 6 μm were cut with a cryostat (CM1900; Leica, Wetzlar, Germany), dried in air, fixed in 95% ethanol for 10 min at − 20 °C, rinsed in PBS, and then incubated with 50-fold serial dilutions of the collected serum samples from experimental mice of each group (*n* = 10) for 60 min at RT. After rinsing in PBS, the cryostat sections were incubated for 60 min with HRP-conjugated goat anti-mouse IgG (ZyMax, CA, USA.; 1:500 dilution) at RT. After washing with PBS, the HRP-binding sites were detected with 0.05% DAB and 0.01% H_2_O_2_.

### Real-time RT-PCR analysis of mRNA expression in testis

The testes from mice (*n* = 5) in four groups were examined. Real-time RT-PCR was performed according to the method described previously [39]. Total RNA was purified from each fresh testis sample using TRIZOL reagent (Invitrogen Corp., Carlabad, CA) according to the manufacturer’s protocol, and its concentration was calculated from the extinction at 260 nm, as determined spectrophotometrically. For the first-strand cDNA synthesis, 10 μg of total RNA was reverse-transcribed with a High Capacity cDNA Archive Kit (PE Applied Biosystems, Foster City, CA, USA) according to the standard protocol. Subsequently, 3 ng of cDNA was amplified by 40 cycles of polymerase chain reaction (PCR) to measure *Ki67*, *Fas*, *FasL*, *caspase8*, *claudin3*, *claudin11*, *occludin*, *ZO1*, *ZO2*, and *GAPDH*, real-time fluorescence-monitored PCR was performed with the Thermal Cycler Dice Real-time System TP800 (TaKaRa) using TaqMan PCR Master Mix Reagents 2× and TaqMan Gene Expression Assays of primers 20×, according to the manufacturer’s instructions. The data was analyzed using Thermal Cycler Dice Real-time System software (TaKaRa) and the comparative C_t_ method (2∆∆C_t_) was used to quantify gene expression levels according to the manufacturer’s instructions. The results were expressed relative to levels of the *GAPDH* transcript used as an internal control. All primers used in this analysis list in Table 2.

**Table 2:** Primers used for real-time RT-PCR

gene	Forward Primer(5′-3′)	Reverse Primer(5′-3′)
*caspase8*	TTGAACAATGAGATCCCCAAA	CCATTTCTACAAAAATTTCAAGCAG
*claudin-3*	TCTCCCAGCCTACGGAGTTA	CAGTTCCCATCTCTCGCTTC
*claudin-11*	TCACAACGTCCACCAATGACTG	GGCACATACAGGAAACCAGATG
*Fas*	GCAGACATGCTGTGGATCTGG	TCACAGCCAGGAGAATCGCAG
*FasL*	TCCAGGGTGGGTCTACTTACTAC	CCCTCTTACTTCTCCGTTAGGA
*GAPDH*	TGTGTCCGTCGTGGATCTGA	TTGCTGTTGAAGTCGCAGGAG
*Ki67*	GCTGTCCTCAAGACAATCATCA	GGCGTTATCCCAGGAGACT
*occludin*	CTTCTGCTTCATCGCTTCC	CTTGCCCTTTCCTGCTTTC
*ZO-1*	ACAAACAGCCCTACCAACC	CCATCCTCATCTTCATCTTCTTC
*ZO-2*	GTTTTTCTTCGTCCTAGTCCC	CATCCATCCCTTCCATCTTTC

### Count the number of epididymal spermatozoa

The epididymal spermatozoa count were determined from both left and right epididymis of mice at day 0 (*n* = 5), day 60 (*n* = 5), day 120 (*n* = 20), and day 150 (*n* = 5) in four groups. Briefly, the epididymides were cut into six pieces and gently pipetted in PBS. The pieces were gently stirred with a pipette, and then passed through a stainless-steel mesh. The epididymal spermatozoa were harvested by centrifugation at 400×*g* for 10 min and resuspended in 5 ml of PBS after washing three times with PBS. The number of epididymal spermatozoa was counted on hemocytometer.

### Statistical analysis

To analyze the differences, ANOVA was used. Statistical significance level was considered at *p*-value < 0.05.

## Results

### TJ107 recovers reproductive functions in TBI mice until day 150 but not day 120

**Fig. 2:**
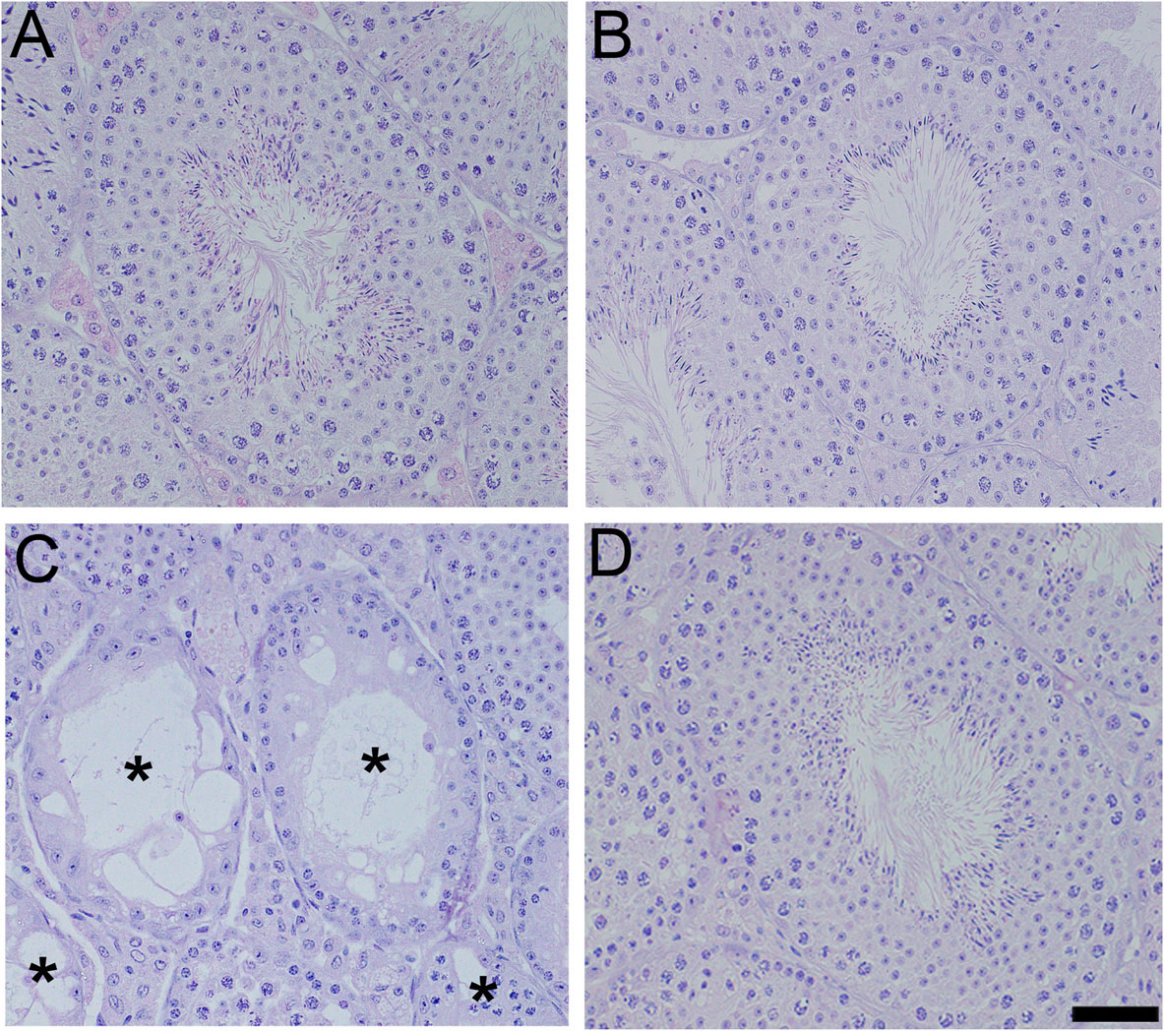
Effect of TJ107 on testes histology after irradiation. Asterisks indicates the damaged seminiferous tubules with azoospermia in Group C **c** at day 120. However, in the Group D **d**, all stages of maturation of the germinal epithelium are seen in normal-appearing seminiferous tubules like in Group A **a** and Group B **b**. Bar = 50 μm

The testicular weight, epididymal spermatozoa, and fertility of mice after the treatment period in four groups are summarized in Table 3. At day 60, in Group C there was significant decreases in body weight, absolute and relative testicular weights, and epididymal spermatozoa counts when compared to those in the Group A. Further, a marginal recovery in body weight and all reproductive parameters were observed at day 120, but on day 150 further decreases in all parameters were noted in this group. Based on histological examinations of the testes (Fig. 2a–d), some atrophic seminiferous tubules with disrupted spermatogenesis were recognized in the testes (Fig. 2c). In sharp contrast, in the Group D, significant recoveries in body weight, epididymal spermatozoa counts, and fertility rates were observed at day 150, although absolute and relative testicular weights did not reach control levels (Table 3). All stages of germinal epithelium maturation, from spermatogonia to spermatozoa were shown in group D in testicular sections showing many normal-appearing seminiferous tubules (Fig. 2d).

**Table 3:** Effect of TBI (6Gy) and TJ107 on testicular weight, epididymal spermatozoa, and fertility in male mice

		Group A	Group B	Group C	Group D
Body weight (g)	Day 0	17.314 ± 0.862	17.314 ± 0.862	17.314 ± 0.862	17.314 ± 0.862
	Day 60	28.501 ± 1.080	28.501 ± 1.080	25.425 ± 1.903*	25.425 ± 1.903*
	Day 120	31.779 ± 2.347	31.932 ± 3.021	28.434 ± 2.669*	28.723 ± 2.289*
	Day 150	33.448 ± 1.118	35.250 ± 3.442	27.662 ± 1.360*	30.060 ± 2.173
Testis weight (g)	Day 0	0.064 ± 0.004	0.064 ± 0.004	0.064 ± 0.004	0.064 ± 0.004
	Day 60	0.097 ± 0.003	0.097 ± 0.003	0.044 ± 0.003*	0.044 ± 0.003*
	Day 120	0.098 ± 0.002	0.102 ± 0.006	0.063 ± 0.006*	0.065 ± 0.004*
	Day 150	0.095 ± 0.004	0.106 ± 0.006	0.057 ± 0.004*	0.069 ± 0.004*
Relative testis weight (%)	Day 0	0.389 ± 0.028	0.389 ± 0.028	0.389 ± 0.028	0.389 ± 0.028
	Day 60	0.354 ± 0.013	0.354 ± 0.013	0.177 ± 0.013*	0.177 ± 0.013*
	Day 120	0.312 ± 0.034	0.315 ± 0.024	0.211 ± 0.016*	0.249 ± 0.806*
	Day 150	0.281 ± 0.012	0.285 ± 0.015	0.191 ± 0.019*	0.225 ± 0.016
spermatozoa (× 10^5^)	Day 0	13.600 ± 0.050	13.600 ± 0.050	13.600 ± 0.050	13.600 ± 0.050
	Day 60	18.600 ± 0.530	18.600 ± 0.530	3.935 ± 0.148*	3.935 ± 0.148*
	Day 120	19.585 ± 1.903	26.332 ± 2.790*	3.987 ± 0.588*	8.860 ± 0.391*
	Day 150	22.560 ± 0.533	25.810 ± 0.871*	3.515 ± 0.548*	23.732 ± 2.041^#^
Rate of Fertility	Day 150	100% (10/10)	100% (10/10)	40% (4/10)*	100% (10/10)^#^

Data are presented as the mean ± standard deviation. Relative testis weight was calculated as a percentage by dividing the combined weight of both testes in milligrams by the body weight in grams. *p < 0.05 vs. Group A; ^**#**^*p* < 0.05 vs. Group C

### TJ107 normalizes proliferation and apoptosis in the testes of 6Gy-treated mice

**Fig. 3:**
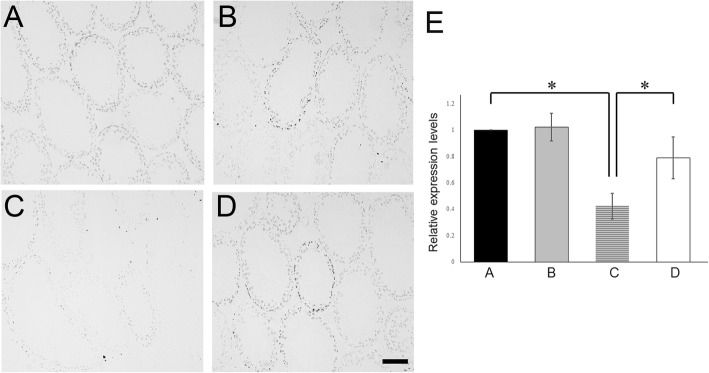
After irradiation and TJ107 treatment shows proliferating cells in the testes. Samples were assessed at day 120 for each group. Immunohistological detection of Ki67 staining in testicular sections in Group A **a**, Group B **b**, Group C **c**, and Group D **d**. Ki67-positive nuclei of proliferating spermatogonia are indicated by dark brown spots, which were detected in almost all seminiferous tubules of the Group A **a**, Group B **b**, and Group D **d** on day 120. In the Group C **c** few seminiferous tubules with Ki67-positive cells were sporadically observed. Bar = 100 μm. **e** Expression of *Ki67* mRNA in the mouse testes of each group at 120 days. Calculation of Relative mRNA intensity was done, and the expression in the control group for each point was set to 1. The data are presented as mean ± standard deviation (*n* = 10). Asterisks indicate *p* < 0.05

**Fig. 4:**
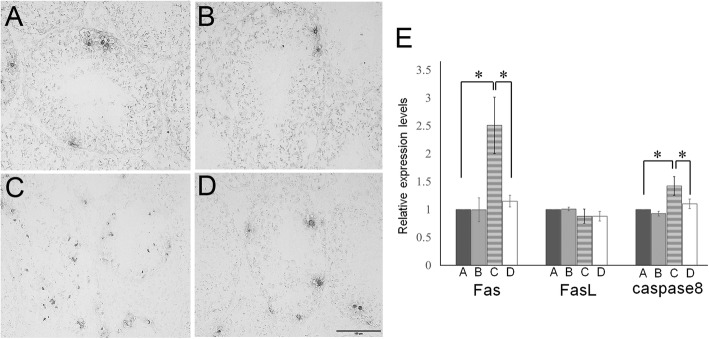
Detection of apoptotic markers in cells of the testes after irradiation and TJ107 treatment. Spermatogenic cell apoptosis was assessed at day 120 in each group. Group A **a**, Group B **b**, Group C **c** and Group D **d** histological detection showed TUNEL staining in the testicular sections. Erratic round-shaped black areas in the seminiferous tubules indicate TUNEL-positive cells. Bar = 100 μm. **e** Expression of *Fas*, Fas ligand (*FasL*), and *caspase8* mRNA in mouse testes of each group at 120 days. Calculation of Relative mRNA intensity was done and the expression in the control group for each point was set to 1. The data are presented as mean ± standard deviation (*n* = 10). Asterisks indicate *p* < 0.05

Next, at day 120, we detected markers of proliferation (Fig. 3a–e) and apoptosis (Fig. 4a–e) in the testes of each group. Few seminiferous tubules with proliferating spermatogonia were detected (Fig. 3c), and increased apoptotic germ cells were detected in atrophic seminiferous tubules (Fig. 4c) were seen in Group C. In the Group D, not only proliferating spermatogonia (Fig. 3d) but also apoptotic germ cells (Fig. 4d) could be detected in the seminiferous tubules, similar to that observed in the Group A (Figs. 3a and 4a). Additionally, the expression of proliferation-related (*Ki67*) and apoptotic (*Fas, FasL,* and *caspase8*) genes in the testicular tissues of each group were examined. Results showed that the expression of *Ki67* was significantly decreased only in the Group C, but not in the other three groups (Fig. 3e). In group C, the expression of *Fas* and *caspase8* were significantly increased but not in the other groups (Fig. 4e). *FasL* level was not significantly altered among the four groups.

### The production of serum anti-germ cell antibodies in 6Gy-treated mice is suppressed by TJ107

**Fig. 5:**
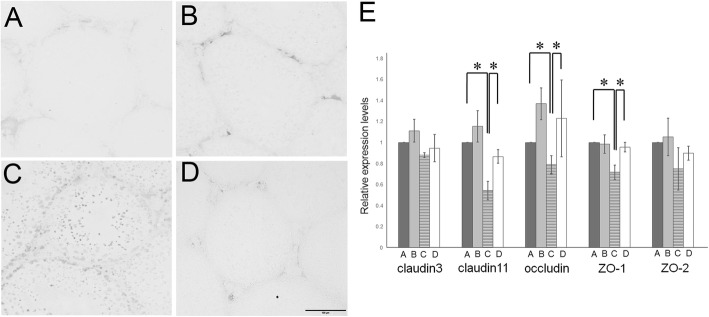
Serum anti-germ cell antibody levels and testes tight junction markers after irradiation and TJ107 treatment. Diluted sera obtained from Group A **a**, Group B **b**, Group C **c** and Group D **d** was reacted with Normal frozen sections of seminiferous tubules which was followed by incubation with HRP-conjugated anti-mouse IgG. Bar = 100 μm. **e** Changes in the mRNA levels of tight-junction associated markers (*claudin3*, *claudin11*, *occludin*, *ZO1*, and *ZO2*) in the mouse testes of each group at 120 days. Calculation of Relative mRNA intensity was done and the expression in the control group for each point was set to 1. The data are presented as mean ± standard deviation (*n* = 10). Asterisks indicate *p* < 0.05

In group C upon assessing the reaction of sera with normal frozen seminiferous tubule section, anti-germ cell antibodies were detected (Fig. 5a–d). Serum autoantibodies preferentially reacted with mature spermatids and spermatozoa in Group C; concurrently, faint immunostaining was detected in the spermatogonia and immature spermatids. Furthermore, to analyze blood-testis-barrier (BTB) function, the expressions of testicular tight junction markers were evaluated by real-time RT-PCR. The mRNA levels of tight junction genes (*claudin11*, *occludin*, and *ZO1*; Fig. 5e) were all significantly increased after TJ107 supplementation. However, *claudin3* and *ZO2* levels were not significantly altered among the four groups.

## Discussion

To preserve the fertility of young male cancer patients, developing methods to minimize or reverse the gonadal toxicity associated with irradiation and chemotherapy is of great importance. Recently, we demonstrated that TJ107, a traditional Japanese medicine, can rescue aspermatogenesis after busulfan treatment in mice [39]. In this study, we evaluated the TJ107efficacy on recovery aspermatogenesis by irradiation. The results showed that disruption of spermatogenesis with a decrease in inter-Sertoli tight junction mRNA levels and a loss of BTB function was induced by 6Gy of TBI which might be related to the production of anti-germ cell antibodies. However, the spermatogenesis was recovered from TBI-induced injuries with TJ107 administration, based on the enhanced expression of tight junction genes and suppression of anti-germ cell antibody production.

In spite of the presence and persistence of undifferentiated spermatogonia appearing to be blocked from further differentiation, variety of testicular toxicant such as irradiation and alkylating agents can damage seminiferous tubule in rodents. In particular, stimulation of spermatogonial differentiation resulting in spermatogenic progression after cancer treatment is because of the suppression of testosterone and gonadotrophin analogs [16, 44]. However, multiple side effects and recovery occurs gradually because of hormone-suppression, to enhance endogenous spermatogenic recovery the application of hormone-suppression treatments has so far been successful in clinical trials [16]. Our results from the present study and our previous work demonstrate that supplementation with TJ107, made of natural products with negligible side effects, can rescue injured reproductive function after not only busulfan but also irradiation treatment.

In these our two studies, we particularly noted that TJ107 in response to busulfan- and irradiation-induced aspermatogenesis has a different therapeutic mechanism. Busulfan-treatment from day 60 to 120 was found to progressively decrease the weight of the testes and the epididymal sperm count, whereas at day 120 after busulfan-treatment TJ107 completely rescued these effects [39]. It was previously shown that busulfan-induced spermatogenic damage results in the upregulation of Toll-like receptor (TLR) 2 and in Sertoli cells and facilitate macrophage infiltration into the testes through TLR4 expression [45]. We first showed that the injured seminiferous epithelium by normalizing macrophage migration can be rescued by TJ107 and reducing the expression of TLR2 and TLR4 [39]. At day 60, in sharp contrast, significant decrease in weight of the testes and epididymal sperm count was induced by irradiation-treatment; at day 120 we observed marginal recovery, and at day 150 further decrease in all parameters was noted. In contrast to the results observed after busulfan-treatment, supplementation with TJ107 significantly recovered epididymal spermatozoa count and fertility rates until day 150 but not day 120 (Table 3). In the present study, causal examination detected that anti-germ cell antibody production and inter-Sertoli tight junction barrier disruption was induced by 6Gy of TBI but this did not cause significant macrophage infiltration into the testes (unpublished observation). TJ107 administration could cure the testicular injuries by reducing the production of serum anti-germ cell antibodies (Fig. 5d) and recovering the tight junctions, as assessed by the observed normalization of *claudin11*, *occludin*, and *ZO1* expression (Fig. 5e). It is well known spermatogenesis is destroyed because of busulfan treatment which directly damages the germ cells and Sertoli cells [46], and in our previous busulfan study, we couldn’t detect any anti-germ cell antibody production (unpublished observation). Accordingly it is also noted that more belated recovery of spermatogenesis is seen in the 6Gy + TJ107 group (day 150) when compared to that in busulfan+TJ107 group (day 120) [39], it can be surmised that autoimmune reaction against germ cells is may affect the delayed recovery of spermatogenesis after irradiation damage to germ cells.

It is well known that BTB is a physical barrier composed of tight junctions between adjacent Sertoli cells and anti-sperm antibody production is enhanced by increase in BTB permeability which causes infertility in males [47–49]. Critical components of the BTB include the integral membrane proteins claudin-11 [47, 48], occluding [50, 51], and the adaptor protein ZO-1 [52, 53] of the tight junction and some reports have shown that with a decrease in ZO-1 and occluding occurs because irradiation-induced BTB disruption [54, 55]. In the present study, with respect to BTB integrity the contribution of claudin, occludin, and ZO-1 (Fig. 5e) was determined in a single dose. It is found that depending on the recovery of the above disorganized tight junctions TBI-induced aspermatogenesis and the recovery of spermatogenesis (Fig. 5e).

Recently, male reproductive functions are widely improved through phytotherapeutic approaches [56]. Tribulus (*Tribulus terrestris* L., Zygophyllaceae) is the main phytotherapics which is promoted to increase testosterone and improve sperm characteristics in humans [57–60], long Jack (*Eurycoma longifolia* Jack, Simaroubaceae) [61, 62], fineleaf fumitory (*Fumaria parviflora* Lam. Ranunculales) [11]., Onion (*Allium cepa* L. Amaryllidaceae) [63] and black seeds *(Nigella sativa* L., Ranunculaceae) [64, 65]. Although, all these evidences based on the therapeutic effects of herbal medicine on male infertility are on animal models and spermatogenic effect on human and its little clinical attestation is yet to be investigated. Thus, we have proposed clinical trial determine the TJ107 effect on reproductive function of oncologic male patients.

We will examine the effects of the other traditional Japanese medicine such as Hachimijiogan and Hochuekkito on oncologic aspermatogenesis and evaluate the efficacy of polyherbal formulation for improving fertility after cancer treatment in next experiments.

## Conclusions

Our studies demonstrated that impaired reproductive function induced by cancer treatments including chemotherapy and radiotherapy related with the different immune-pathophysiological conditions can be cured by TJ107; therefore, we conclude that the administration of TJ107 is potentially useful for male cancer patients. In future experiments, the effects of the other traditional Japanese medicines such as Hachimijiogan and Hochuekkito, which have been routinely administered to patients with male infertility, on cancer treatments-induced testicular injuries will be examined.

## Data Availability

The datasets used and analysed during the current study are available from the corresponding author on reasonable request.

## Abbreviations

BTB: blood-testis-barrier
TBI: total body irradiation
TJ107: Goshajinkigan
TLR: Toll-like receptor
